# Safety and Efficacy of Different Surgical Sequences for Endovascular Aortic Repair and Percutaneous Coronary Intervention

**DOI:** 10.3390/jcm14227962

**Published:** 2025-11-10

**Authors:** Yuping Lei, Dongfeng Zhang, Jinfan Tian, Nan Nan, Mingduo Zhang, Yuguo Xue, Min Zhang, Yuan Zhou, Xiantao Song, Hongjia Zhang

**Affiliations:** 1 Department of Cardiology, Beijing Anzhen Hospital, Capital Medical University, Beijing 100029, China; fantaray@163.com (Y.L.); dongfengdoctor@outlook.com (D.Z.); tjfbama@163.com (J.T.); 13910096973@139.com (N.N.); wodexuexi@126.com (M.Z.); xueyuguo2006@163.com (Y.X.); minjun1977@sina.com (M.Z.); zhouyuanfw1019@sina.com (Y.Z.); 2 Department of Cardiology, Liaocheng People’s Hospital, Liaocheng 252000, China; 3 Department of Cardiovascular Surgery, Beijing Anzhen Hospital, Capital Medical University, Beijing 100029, China; zhanghongjia722@ccmu.edu.cn

**Keywords:** simultaneous surgery, coronary artery disease, aortic aneurysm, endovascular aortic repair, percutaneous coronary intervention

## Abstract

**Objective:** This study is designed to systematically assess the safety and efficacy profiles associated with varying procedural sequences of endovascular aortic repair (EVAR) and percutaneous coronary intervention (PCI) in clinical practice. **Methods:** We conducted a retrospective cohort analysis encompassing patients diagnosed with aortic aneurysm and concomitant coronary artery disease (CAD) who underwent EVAR at Beijing Anzhen Hospital, Capital Medical University, between January 2010 and December 2022, with planned staged (preoperative or postoperative) or simultaneous PCI. The cohort was stratified into three groups: PCI followed by EVAR, EVAR followed by PCI, and simultaneous EVAR and PCI. The primary endpoint was a composite of all-cause mortality, non-fatal myocardial infarction, cerebrovascular events, and aortic-related complications within 12 months post-intervention. Secondary endpoints included duration of hospital stay, total hospitalization costs, and incidence of in-hospital adverse events. Multivariate logistic regression analysis was employed to identify independent predictors of the primary endpoint. **Results:** The study cohort comprised 374 patients, with 209 (55.9%) undergoing PCI followed by EVAR, 133 (35.6%) receiving EVAR followed by PCI, and 32 (8.5%) undergoing simultaneous EVAR and PCI. Comparative analysis revealed no statistically significant differences in the incidence of in-hospital adverse events (*p* = 0.867) or the risk of primary endpoint events (*p* = 0.645) across the three treatment groups. Notably, the simultaneous treatment group demonstrated a significantly reduced total hospital stay (10.6 days) compared to the PCI followed by EVAR group (16.0 days) and the EVAR followed by PCI group (17.2 days) (*p* < 0.001), accompanied by lower hospitalization costs (*p* = 0.002). **Conclusions:** For patients with aortic aneurysm complicated by CAD requiring both EVAR and PCI, simultaneous intervention appears to be a safe and feasible therapeutic option. This approach significantly reduces hospital stay duration and associated costs without increasing the risk of in-hospital adverse events or compromising 12-month postoperative outcomes. However, this exploratory finding requires validation in large-scale randomized controlled trials.

## 1. Introduction

The management of patients presenting with concomitant aortic aneurysm and coronary artery disease (CAD) represents a complex clinical challenge in cardiovascular medicine. Aortic aneurysm, characterized by pathological dilation of the aortic wall, carries a substantially elevated risk of rupture when the thoracic aortic diameter exceeds 5.5 cm or the abdominal aortic diameter surpasses 5 cm [1,2]. Endovascular Aneurysm Repair (EVAR), a minimally invasive interventional technique involving the deployment of a covered stent within the aortic lumen, has emerged as the preferred treatment modality for most aortic pathologies due to its reduced trauma, accelerated recovery, and lower complication rates compared to conventional open surgical approaches [3]. CAD, a critical comorbidity in this patient population, significantly contributes to both perioperative and long-term mortality, accounting for 60–70% and 40–70% of deaths, respectively [4,5]. The management of significant coronary artery stenosis typically involves revascularization through either Percutaneous Coronary Intervention (PCI) or Coronary Artery Bypass Grafting (CABG). Epidemiological studies indicate that the prevalence of concomitant aortic aneurysm and CAD ranges from 40% to 55%, with the co-occurrence of abdominal aortic aneurysm (AAA) and CAD reaching 31% to 90% [6]. This substantial comorbidity burden necessitates the development of comprehensive therapeutic strategies.

Current evidence regarding the comparative efficacy of sequential strategies for EVAR and PCI remains limited. In clinical practice, most medical institutions adopt a staged surgical approach for patients presenting with concurrent aortic aneurysms and CAD, wherein coronary revascularization is performed either preceding or following EVAR [7,8]. However, staged surgery presents several limitations. Primarily, this approach carries an elevated risk of perioperative complications: prioritizing CAD management may predispose patients to aneurysm rupture during the interoperative interval, whereas addressing aortic aneurysm repair first may increase the likelihood of perioperative myocardial infarction. Furthermore, the requirement for multiple hospitalizations and surgical procedures not only imposes significant psychological and financial burdens on patients but also potentially leads to inefficient utilization of healthcare resources. Moreover, perioperative management in staged surgery presents increased complexity, particularly in the transition between antiplatelet and anticoagulation therapies, which may elevate the risk of hemorrhagic or thrombotic complications.

Although simultaneous intervention theoretically offers advantages, including reduced procedural complications, decreased hospitalization frequency, shortened hospital stays, and cost-effectiveness, this approach introduces greater surgical complexity, and its safety profile requires further validation. Preliminary observational studies have demonstrated the safety and feasibility of concurrent PCI and EVAR for managing concomitant aortic and coronary pathologies [9,10]; however, these investigations lacked direct comparative analysis with staged procedures. Weng et al. reported that optimized perioperative management and reduced interprocedural intervals between PCI and EVAR could achieve postoperative outcomes comparable to standard EVAR without exacerbating severe CAD risks [11]. Notably, their “fast-track management” protocol involved performing the procedures within a compressed timeframe (one month or two weeks) rather than simultaneously. Consequently, further research is warranted to establish the optimal surgical sequence for EVAR and PCI interventions.

As a leading institution in cardiovascular research, Beijing Anzhen Hospital, affiliated with Capital Medical University and designated as the National Clinical Research Center for Cardiovascular Diseases, possesses substantial expertise in the management of aortic diseases and CAD. The center has developed comprehensive therapeutic protocols for EVAR and coronary revascularization, including preliminary comparative analyses of surgical outcomes. Building upon this foundation, this investigation seeks to systematically evaluate the safety and efficacy profiles of various surgical sequence strategies, with particular emphasis on concurrent EVAR and PCI, in patients presenting with concomitant aortic aneurysm and CAD. The findings aim to provide evidence-based guidance for clinical decision-making and therapeutic management of this complex patient population.

## 2. Materials and Methods

### 2.1. Study Design and Population

**Figure 1:**
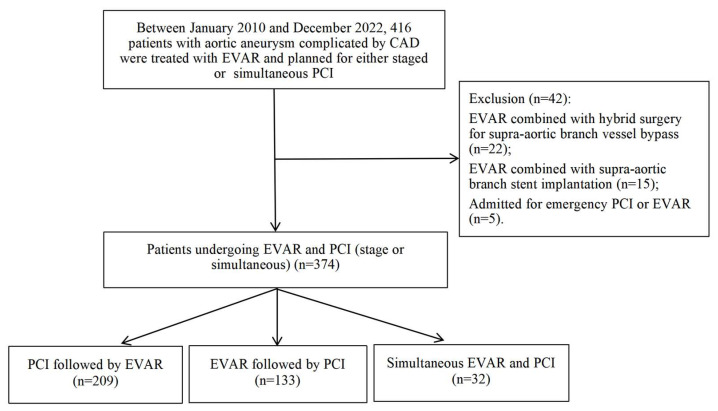
The Flowchart of Patient Selection for the Study.

This retrospective cohort study was conducted at Beijing Anzhen Hospital, Capital Medical University, from January 2010 to December 2022 (Figure 1). The study population comprised 416 patients with aortic aneurysm complicated by CAD who underwent EVAR with planned staged (preoperative or postoperative) or simultaneous PCI. All patients routinely underwent preoperative coronary artery evaluation, with coronary computed tomography angiography (CCTA) typically being the preferred method. In cases where CCTA findings were positive, or when encountering conditions such as severe coronary calcification, elevated heart rate, or suboptimal image quality, coronary angiography was further performed. Exclusion criteria were applied to 22 patients who underwent EVAR combined with hybrid surgery for supra-aortic branch vessel bypass, 15 patients who underwent EVAR with supra-aortic branch stent implantation, and 5 patients admitted for emergency PCI or EVAR, resulting in a final cohort of 374 eligible patients.

The treatment sequence was determined through multidisciplinary team evaluation involving cardiology, cardiovascular surgery, and anesthesiology specialists, categorizing patients into three groups: (1) 209 patients underwent PCI followed by EVAR, with PCI performed to optimize myocardial perfusion and EVAR conducted during the same or subsequent hospitalization; (2) 133 patients underwent EVAR followed by PCI, with EVAR performed to address the aortic aneurysm and PCI conducted after patient stabilization; (3) 32 patients underwent simultaneous EVAR and PCI within a single anesthesia session. All procedures were performed by specialized vascular surgery and cardiology teams following standardized protocols.

### 2.2. Study Endpoints and Definitions

This study systematically collected and analyzed comprehensive patient data, encompassing demographic characteristics, clinical profiles, surgical procedures, and perioperative parameters. The study variables incorporated fundamental patient attributes (age, gender, smoking history, body mass index [BMI]), comorbidities (hypertension, hyperlipidemia, diabetes), cardiovascular risk factors (family history of CAD, previous myocardial infarction), and functional indicators (glomerular filtration rate [GFR], left ventricular ejection fraction [LVEF]). Additionally, disease-specific characteristics were evaluated, including CAD classification, aortic aneurysm manifestations (symptomatology, diameter, anatomical location), and other relevant clinical markers. Surgical and perioperative metrics comprised coronary lesion quantification and distribution, number of stents implanted, temporal intervals between EVAR and PCI, duration of hospitalization, and associated healthcare expenditures. Data acquisition was conducted through medical record reviews and patient follow-ups.

A standardized 12-month follow-up period was implemented post-discharge to minimize temporal bias in outcome assessment. Follow-up data were collected through structured outpatient visits and telephone interviews to ensure data integrity. The primary endpoint was a composite of all-cause mortality, non-fatal myocardial infarction, stroke, and aortic-related adverse events (including aneurysm expansion, stent displacement, or all types of endoleak) within the 12-month follow-up period. Secondary endpoints encompassed hospital length of stay, hospitalization costs, and in-hospital adverse events. In-hospital adverse events were defined as any complications that occurred during the index or subsequent hospitalizations, including but not limited to in-hospital mortality, aortic aneurysm rupture, acute myocardial infarction, acute heart failure, cerebral infarction, acute kidney injury, infection, bleeding, type I endoleak, type II endoleak, spinal cord ischemia, vascular access complications, intensive care unit (ICU) admission, and ventilator requirement.

### 2.3. Surgical Protocol

#### 2.3.1. Staged Surgical Intervention

The surgical sequence was determined according to the severity of CAD and aortic stability, aiming to optimize the balance between myocardial ischemia risk and aortic aneurysm rupture. PCI was performed through radial artery access, followed by coronary angiography (CAG) and subsequent coronary balloon angioplasty or drug-eluting stent implantation. Intraoperative heparinization (100 U/kg) was administered to maintain activated clotting time (ACT) between 250–350 s, with dual antiplatelet therapy (aspirin 100 mg daily and clopidogrel 75 mg daily or ticagrelor 90 mg twice daily) continued for 6–12 months postoperatively. EVAR was conducted under general anesthesia, with a covered stent deployed via the femoral artery approach. Intraoperative angiography confirmed proper stent positioning and assessed for endoleak, with heparinization protocols identical to those used in PCI.

#### 2.3.2. Simultaneous Surgical Intervention

EVAR and PCI were performed concurrently under general anesthesia, with critical technical considerations including: (1) controlled contrast agent administration to minimize the risk of contrast-induced nephropathy; (2) heparinization to maintain ACT within 250–350 s, ensuring optimal anticoagulation while mitigating bleeding risk; and (3) precise stent positioning achieved through dual-catheter angiography (utilizing a pigtail catheter in the ascending aorta and the stent delivery system’s contrast channel) to ensure accurate alignment with renal artery ostia and iliac artery branches.

#### 2.3.3. Standardized Perioperative Management

All surgical groups adhered to a standardized perioperative protocol. Intraoperative monitoring included continuous assessment of invasive arterial pressure, central venous pressure, and myocardial injury markers, with real-time evaluation of myocardial perfusion and stent apposition. Hemodynamic control was maintained with systolic blood pressure between 100–120 mmHg and heart rate between 60–80 beats per minute, achieved through intravenous β-blockers (e.g., metoprolol) combined with sodium nitroprusside to prevent aortic aneurysm rupture or endoleak due to blood pressure fluctuations. Anticoagulation and hemostasis protocols involved weight-adjusted heparinization during both PCI and EVAR, with vascular closure devices (e.g., ProGlide suture) employed at the femoral artery puncture site postoperatively to reduce bleeding complications.

### 2.4. Statistical Analysis

Continuous variables were initially assessed for normality using appropriate statistical tests. Variables demonstrating normal distribution are presented as the mean ± standard deviation, with intergroup comparisons conducted using independent samples t-test. For variables exhibiting non-normal distributions or unequal variances, the Kruskal–Wallis H test was employed. Categorical variables are expressed as frequencies (percentages), and intergroup comparisons were performed using the chi-squared test, with Fisher’s exact test applied when the expected frequency was <5. The logistic regression model was employed to identify independent predictors of composite endpoint events. Variable selection was based on both clinical relevance and the results of univariate analysis. Variables with potential prognostic significance in aortic aneurysm and CAD (e.g., age, sex, diabetes mellitus, number of coronary lesions) were included in the multivariate logistic regression analysis when they showed statistical significance (*p* < 0.05) in the univariate logistic regression analysis. In addition, since the surgical method is a key variable in our study, we have also included the surgical method in the multivariate logistic regression analysis. All statistical analyses were executed using SPSS version 20.0 (SPSS Inc., Chicago, IL, USA), with a two-tailed *p*-value <0.05 considered statistically significant.

## 3. Results

### 3.1. Baseline Clinical Characteristics of Patients

From January 2010 to December 2022, a cohort of 374 patients who underwent EVAR and PCI at Beijing Anzhen Hospital, Capital Medical University, was retrospectively analyzed. Patients were stratified into three groups based on the temporal sequence of EVAR and PCI: 209 patients (55.9%) underwent PCI followed by EVAR, 133 patients (35.6%) underwent EVAR followed by PCI, and 32 patients (8.5%) underwent simultaneous EVAR and PCI.

Table 1 summarizes the baseline characteristics of the study population. While the three groups exhibited generally comparable baseline profiles, significant differences were observed in specific clinical parameters. Male patients predominated across all groups, with a significantly higher proportion in the PCI followed by EVAR group (94.3%) compared to the EVAR followed by PCI group (86.5%) and the simultaneous group (90.6%) (*p* = 0.046). Hyperlipidemia prevalence was notably higher in the simultaneous group (56.3%) than in the PCI followed by EVAR group (35.3%) and the EVAR followed by PCI group (31.1%) (*p* = 0.021). The EVAR followed by PCI group demonstrated a significantly higher prevalence of diabetes (47.4%, *p* < 0.001). Additionally, a history of myocardial infarction was more prevalent in the PCI followed by EVAR group (26.8%) and the simultaneous group (25.0%) compared to the EVAR followed by PCI group (15.0%) (*p* = 0.037). The mean age across all groups was approximately 68 years. No significant intergroup differences were observed in smoking history, BMI, hypertension prevalence, GFR, LVEF, CAD classification, aortic aneurysm symptoms, aneurysm diameter, or anatomical location.

### 3.2. CAG and PCI Outcomes

The findings from CAG and PCI are presented in Table 2. The distribution of coronary artery lesions, encompassing the left main coronary artery (LM), left anterior descending artery (LAD), left circumflex artery (LCX), and right coronary artery (RCA), demonstrated no statistically significant differences across the study groups (*p* > 0.05). Notably, the concurrent treatment group exhibited a significantly higher prevalence of three-vessel disease (40.6%) compared to the other groups (*p* < 0.05). Furthermore, the PCI followed by EVAR group required a significantly greater number of stents during PCI (*p* < 0.001), suggesting the necessity for more extensive revascularization to ensure optimal hemodynamic stability prior to the subsequent EVAR procedure.

### 3.3. Endpoint Events Analysis

As presented in Table 3, the distribution of endpoint events among the surgical intervention groups was systematically analyzed. The concurrent treatment cohort exhibited a statistically significant reduction in mean hospital stay duration (10.6 days) when compared to both the PCI followed by EVAR group (16.0 days) and the EVAR followed by PCI group (17.2 days) (*p* < 0.001). Furthermore, a significant reduction in hospitalization costs was observed in the concurrent treatment group (*p* = 0.002), suggesting potential economic benefits and enhanced patient compliance associated with simultaneous surgical intervention.

Within the study population, 144 patients (38.5%) experienced adverse events during the hospitalization period, including mortality (*n* = 2), aortic aneurysm rupture (*n* = 1), acute myocardial infarction (*n* = 14), acute heart failure (*n* = 6), cerebral infarction (*n* = 5), acute kidney injury (*n* = 9), infection (*n* = 35), hemorrhage (*n* = 9), type I endoleak (*n* = 7), type II endoleak (*n* = 8), ICU admission (*n* = 85), and ventilator requirement (*n* = 47). Statistical analysis revealed no significant intergroup differences in adverse event incidence (*p* = 0.867).

Regarding the primary endpoint, the analysis demonstrated no statistically significant differences among the treatment groups (*p* = 0.795), with incidence rates of 9.6% in the PCI followed by EVAR group, 8.3% in the EVAR followed by PCI group, and 6.3% in the concurrent EVAR and PCI group.

### 3.4. Risk Factor Analysis for the Primary Endpoint in Patients Undergoing EVAR and PCI

As detailed in Table 4, both univariate and multivariate logistic regression analyses were conducted to assess risk factors associated with the primary endpoint. The univariate analysis initially revealed that the surgical sequence strategy was not significantly associated with the primary endpoint risk (*p* = 0.797). Considering the surgical method is a key variable in our study, however, we have also included the surgical method in the multivariate logistic regression analysis, and the multivariate analysis confirmed that simultaneous EVAR and PCI did not increase the risk of primary endpoint events (*p* = 0.645). In addition, a history of myocardial infarction was identified as a significant predictor, with the univariate analysis indicating an elevated risk (OR = 2.472, 95% CI: 1.173–5.210, *p* = 0.017), which remained significant in the multivariate analysis (OR = 2.727, 95% CI: 1.129–6.587, *p* = 0.026). Although the univariate analysis suggested that LAD artery lesions might be a risk factor (OR = 3.975, 95% CI: 1.186–13.330, *p* = 0.025), this association did not achieve statistical significance in the multivariate analysis (OR = 2.008, 95% CI: 0.491–8.211, *p* = 0.332). Triple-vessel disease was significantly associated with increased risk in both univariate (OR = 6.273, 95% CI: 2.065–19.058, *p* = 0.001) and multivariate analyses (OR = 4.576, 95% CI: 1.240–16,889, *p* = 0.022). The number of stents emerged as a critical predictor, with the univariate analysis showing an increased risk (OR = 2.056, 95% CI: 1.477–2.863, *p* < 0.001), which was further corroborated in the multivariate analysis (OR = 2.553, 95% CI: 1.695–3.847, *p* < 0.001). Additionally, in-hospital adverse events were significantly associated with the primary endpoint in the univariate analysis (OR = 4.182, 95% CI: 1.927–9.076, *p* < 0.001), and this association persisted in the multivariate analysis (OR = 5.998, 95% CI: 2.401–14.983, *p* < 0.001).

## 4. Discussion

This study represents the first comprehensive investigation into the safety and efficacy of simultaneous EVAR and PCI in patients presenting with both aortic aneurysm and CAD. Comparative analysis of perioperative adverse events revealed no statistically significant differences between the simultaneous intervention group and the staged surgery groups, suggesting that the simultaneous approach does not elevate perioperative risk. Notably, the simultaneous strategy demonstrated significant advantages in reducing both hospital length of stay and overall medical costs compared to staged procedures. Twelve-month follow-up data further substantiated the long-term reliability of simultaneous intervention, with no significant differences observed in primary endpoint event rates across the three treatment groups. These findings indicate that simultaneous EVAR and PCI offers substantial value in process optimization and may emerge as a preferred therapeutic strategy for this patient population.

Quantitative analysis of healthcare resource utilization revealed that the simultaneous intervention group exhibited a mean hospital stay of 10.6 days, significantly shorter than both the PCI-first group (16.0 days) and the EVAR-first group (17.2 days). In the context of high bed turnover rates characteristic of large tertiary hospitals in China, this reduction in hospitalization duration could effectively alleviate bed pressure in cardiovascular surgery and cardiology departments, while simultaneously reducing per capita medical expenditures and costs associated with postoperative complication prevention. Furthermore, total hospitalization costs were significantly lower in the simultaneous intervention group. These findings align with previous research on simultaneous open surgical procedures, which have similarly demonstrated the economic advantages of this approach. For instance, Williams et al. [12], through retrospective analysis, established that simultaneous abdominal aortic aneurysm repair combined with CABG not only streamlined patient management but also reduced the healthcare burden associated with multiple hospitalizations and surgical interventions.

The incidence of in-hospital adverse events demonstrated no significant differences across the three treatment groups, thereby substantiating the safety profile of simultaneous EVAR and PCI. Notably, even in cases of complex cardiovascular disease, the simultaneous surgical approach did not significantly elevate the risk of adverse events, including acute myocardial infarction, stroke, or acute heart failure. These findings align with previous investigations into simultaneous aortic and coronary interventions [13,14,15]. Specifically, Okada et al. reported that concurrent CABG with total aortic arch replacement did not increase in-hospital mortality, even among older patients with more severe comorbidities, thereby reinforcing the efficacy of simultaneous aortic and coronary procedures in complex cases [13]. Furthermore, Pecoraro et al. demonstrated that early EVAR following PCI did not elevate the incidence of postoperative adverse events, further validating the safety and feasibility of performing both procedures within a single hospitalization [16].

The 12-month follow-up data revealed no significant differences in the risk of primary endpoint occurrence among the three groups: 9.6% in the PCI followed by EVAR group, 8.3% in the EVAR followed by PCI group, and 6.3% in the concurrent treatment group. Multivariate regression analysis confirmed that simultaneous EVAR and PCI did not increase the risk of primary endpoint occurrence. Although not statistically significant, the concurrent treatment group exhibited a relatively lower risk of primary endpoint events, suggesting that simultaneous management may offer more comprehensive cardiovascular protection. This observation is supported by a previous retrospective study involving seven patients who underwent concurrent EVAR and PCI, which reported complete resolution or significant alleviation of clinical symptoms, improved quality of life, and absence of reoperation or mortality [17]. The early to mid-term outcomes of that study are consistent with the findings of the present investigation, further corroborating the safety and efficacy of concurrent EVAR and PCI.

The present investigation employed both univariate and multivariate logistic regression analyses, which identified a history of myocardial infarction, three-vessel CAD, the number of stents implanted, and adverse in-hospital events as independent risk factors for primary endpoint events. These findings underscore the critical importance of comprehensive preoperative cardiac evaluation and risk stratification in patients undergoing complex surgical procedures. Consequently, meticulous assessment of individual patient risk profiles and comorbidities is essential for determining optimal therapeutic strategies, while perioperative management protocols should be refined to minimize complications and enhance medium- to long-term clinical outcomes [18]. Enhanced interdisciplinary collaboration among cardiologists, vascular surgeons, and anesthesiologists is particularly crucial in cases requiring simultaneous interventions, as this approach may optimize patient prognosis [19,20]. Although the strategy of managing both conditions within a single hospitalization demonstrates potential for reducing patient burden and healthcare system costs, its clinical implementation necessitates continuous evaluation and refinement. Through further research and strengthened multidisciplinary cooperation, treatment outcomes for patients presenting with concomitant aortic aneurysm and CAD can be substantially improved.

Future research should focus on optimizing the technical processes of concurrent surgical procedures and perioperative management strategies. This includes exploring techniques to minimize the use of contrast agents and determining the optimal duration of postoperative antiplatelet therapy. Additionally, it is necessary to establish standardized diagnostic and treatment pathways and risk stratification models. These will assist clinicians in more accurately selecting patients suitable for concurrent surgical interventions and facilitate the formulation of personalized treatment regimens. The potential applications of this study not only contribute to improving clinical outcomes and expanding therapeutic options but also offer important references for the optimal allocation of medical resources.

This study presents several notable limitations that warrant consideration. Firstly, the retrospective design inherently introduces potential selection bias due to risk-based surgical decisions. The limited and unequal sample size, especially the smaller simultaneous group (*n* = 32), potentially reduces the statistical power of the present study. In clinical practice, the preferential selection of patients with reduced surgical risk for simultaneous EVAR and PCI may result in systematic disparities in baseline risk profiles between the concurrent and staged treatment groups. Such selection bias could potentially overestimate the safety profile of concurrent interventions or obscure postoperative complication risks, thereby compromising the internal validity of the study outcomes. Secondly, the 12-month follow-up duration may be inadequate for evaluating long-term clinical endpoints and would underestimate long-term complications.

Future investigations should prioritize large-scale prospective studies to validate these findings, incorporating extended follow-up periods to comprehensively assess the comparative impact of concurrent versus staged interventions on patient outcomes. The implementation of randomized group allocation and the accumulation of large-scale clinical data will enable more robust validation of the efficacy and safety profiles of simultaneous procedures, while facilitating the precise identification of patient subgroups most likely to derive clinical benefit from this therapeutic approach.

## 5. Conclusions

The simultaneous implementation of EVAR and PCI appears to be a safe and feasible therapeutic option for patients with concurrent aortic aneurysm and CAD. This integrated approach significantly reduces the duration of hospital stay and healthcare costs while maintaining cardiovascular safety profiles comparable to those of traditional staged surgical interventions. The study provides valuable insights for clinical application and contributes to the management of such cardiovascular patients. However, this conclusion remains an exploratory finding that requires validation through larger-scale, prospective, randomized controlled trials to establish definitive clinical evidence.

## Figures and Tables

**Table 1:** Comparison of Baseline Characteristics Among Patients in Different Surgical Groups.

Characteristics	PCI Followed by EVAR Group (*n* (*n*%))	EVAR Followed by PCI Group (*n* (*n*%))	Simultaneous EVAR and PCI Group (*n* (*n*%))	*p*-Value
Gender				0.046
Male	197 (94.3)	115 (86.5)	29 (90.6)	
Female	12 (5.7)	18 (13.5)	3 (9.4)	
Age (years) [Mean (SD)]	67.7 (8.6)	68.1 (9.2)	67.5 (8.2)	0.895
Smoking History	141 (67.5)	90 (67.7)	21 (65.6)	0.975
BMI [Mean (SD)]	25.6 (3.2)	25.6 (3.7)	25.5 (4.3)	0.978
Hypertension	155 (74.2)	95 (71.4)	24 (75.0)	0.834
Hyperlipidemia	65 (31.1)	47 (35.3)	18 (56.3)	0.021
Diabetes Mellitus	51 (24.4)	63 (47.4)	6 (18.8)	<0.001
Family History of CAD	65 (31.1)	50 (37.6)	7 (21.9)	0.183
History of Myocardial Infarction	56 (26.8)	20 (15.0)	8 (25.0)	0.037
GFR (ml/min) [Mean (SD)]	94.1 (28.6)	94.7 (33.3)	92.9 (28.4)	0.957
LVEF (%) [Mean (SD)]	57.9 (6.9)	57.6 (8.0)	56.5 (7.8)	0.607
CAD Classification				0.145
Stable Angina	60 (28.7)	26 (19.5)	4 (12.5)	
Unstable Angina	138 (66.0)	97 (72.9)	25 (78.1)	
Acute Myocardial Infarction	11 (5.3)	10 (7.5)	3 (9.4)	
Aneurysm Symptoms	37 (17.7)	30 (22.6)	7 (21.9)	0.521
Aneurysm Diameter (mm) [Mean (SD)]	63.5 (7.3)	63.4 (7.0)	62.3 (7.4)	0.687
Aneurysm Classification				0.473
Thoracic Aortic Aneurysm	46 (22.0)	37 (27.8)	8 (25.0)	
Abdominal Aortic Aneurysm	163 (78.0)	96 (72.2)	24 (75.0)	

**Table 2:** Comparison of CAG and PCI Outcomes Among Different Surgical Groups.

Characteristics	PCI Followed by EVAR Group (*n* (*n*%))	EVAR Followed by PCI Group (*n* (*n*%))	Simultaneous EVAR and PCI Group (*n* (*n*%))	*p*-Value
Coronary Lesion Distribution				
LM	18 (8.6)	8 (6.0)	3 (9.4)	0.639
LAD	155 (74.2)	95 (71.4)	24 (75.0)	0.834
LCX	117 (56.0)	78 (58.6)	23 (71.9)	0.235
RCA	142 (67.9)	103 (77.4)	24 (75.0)	0.150
Number of Coronary Lesions				0.216
Single-Vessel	59 (28.2)	31 (23.3)	6 (18.8)	
Two-Vessel	103 (49.3)	64 (48.1)	13 (40.6)	
Three-Vessel	47 (22.5)	38 (28.6)	13 (40.6)	
Number of Stents Implanted	2.11 (1.0)	1.7 (0.6)	1.9 (0.9)	<0.001

**Table 3:** Comparison of Endpoint Events Among Different Surgical Groups.

Characteristics	PCI Followed by EVAR Group (*n* = 209)	EVAR Followed by PCI Group (*n* = 133)	Simultaneous EVAR and PCI Group (*n* = 32)	*p*-Value
Time Interval Between Two Operations (days) [Mean (SD)]	12.9 (10.3)	11.1 (9.4)		0.100
Hospital Stay (days) [Mean (SD)]	16.0 (6.0)	17.2 (5.8)	10.6 (3.3)	<0.001
Hospitalization Costs (RMB) [Mean (SD)]	243,508.5 (68,146.8)	253,694.6 (70,893.8)	205,325.2 (68,992.7)	0.002
In-hospital Adverse Events	78 (37.3)	53 (39.8)	13 (40.6)	0.867
The Primary Endpoint of 12-month Follow-up	20 (9.6)	11 (8.3)	2 (6.3)	0.795

**Table 4:** Univariate and Multivariate Logistic Regression Analysis for Risk Factors Associated with the Primary Endpoint.

Characteristics	Univariate Analysis		Multivariate Analysis	
	OR (95% CI)	*p*-Value	OR (95% CI)	*p*-Value
Surgical Method		0.797		0.645
PCI Followed by EVAR Group	1		1	
EVAR Followed by PCI Group	0.852 (0.394–1.840)	0.684	1.320 (0.513–3.399)	0.565
Simultaneous EVAR and PCI Group	0.630 (0.140–2.834)	0.547	0.620 (0.120–3.201)	0.568
Gender		0.561		
Female	1			
Male	1.550 (0.354–6.787)			
Age (years)	1.010 (0.970–1.053)	0.619		
Smoking History		0.494		
No	1			
Yes	1.322 (0.595–2.937)			
BMI	0.935 (0.842–1.038)	0.206		
Hypertension		0.250		
No	1			
Yes	1.720 (0.688–4.297)			
Hyperlipidemia		0.335		
No	1			
Yes	1.429 (0.692–2.953)			
Diabetes Mellitus		0.348		
No	1			
Yes	1.421 (0.682–2.964)			
Family History of CAD		0.766		
No	1			
Yes	0.889 (0.409–1.932)			
History of Myocardial Infarction		0.017		0.026
No	1		1	
Yes	2.472 (1.173–5.210)		2.727 (1.129–6.587)	
GFR (ml/min)	0.995 (0.983–1.007)	0.418		
LVEF (%)	1.009 (0.970–1.050)	0.645		
CAD Classification		0.802		
Stable Angina	1			
Unstable Angina	0.947 (0.406–2.211)	0.901		
Acute Myocardial Infarction	1.464 (0.357–6.003)	0.596		
Aneurysm Symptoms		0.809		
No	1			
Yes	0.892 (0.354–2.247)			
Aneurysm Diameter (mm) [Mean (SD)]	1.012 (0.962–1.065)	0.637		
Aneurysm Classification		0.205		
Thoracic Aortic Aneurysm	1			
Abdominal Aortic Aneurysm	0.529 (0.198–1.414)			
Coronary Lesion Distribution				
LM		0.704		
No	1			
Yes	0.750 (0.170–3.306)			
LAD		0.025		0.332
No	1		1	
Yes	3.975 (1.186–13.330)		2.008 (0.491–8.211)	
LCX		0.083		
No	1			
Yes	2.021 (0.912–4.477)			
RCA		0.191		
No	1			
Yes	1.841 (0.737–4.596)			
Number of Coronary Lesions		<0.001		0.001
Single-Vessel	1		1	
Two-Vessel	1.070 (0.314–3.648)	0.914	0.788 (0.200–3.099)	0.733
Three-Vessel	6.273 (2.065–19.058)	0.001	4.576 (1.240–16,889)	0.022
Number of Stents Implanted	2.056 (1.477–2.863)	<0.001	2.553 (1.695–3.847)	<0.001
In-hospital Adverse Events		<0.001		<0.001
None	1		1	
Yes	4.182 (1.927–9.076)		5.998 (2.401–14.983)	

## Data Availability

The datasets generated during the present study are available upon reasonable request from the corresponding author.

## Abbreviations

The following abbreviations are used in this manuscript:

EVAR	Endovascular aortic repair
PCI	Percutaneous coronary intervention
CAD	Coronary artery disease
CABG	Coronary artery bypass grafting
CCTA	Coronary computed tomography angiography
BMI	Body mass index
GFR	Glomerular filtration rate
LVEF	Left ventricular ejection fraction
ICU	Intensive care unit
CAG	Coronary angiography
ACT	Activated clotting time
LM	Left main coronary artery
LAD	Left anterior descending artery
LCX	Left circumflex artery
RCA	Right coronary artery

## References

[1] Haque K., Bhargava P. (2022). Abdominal Aortic Aneurysm. Am. Fam. Physician.

[2] Salmon M. (2022). NADPH Oxidases in Aortic Aneurysms. Antioxidants.

[3] Mostafa K., Pfarr J., Langguth P., Schäfer J.P., Trentmann J., Koktzoglou I., Edelman R.R., Bueno Neves F., Graessner J., Both M. (2022). Clinical Evaluation of Non-Contrast-Enhanced Radial Quiescent-Interval Slice-Selective (QISS) Magnetic Resonance Angiography in Comparison to Contrast-Enhanced Computed Tomography Angiography for the Evaluation of Endoleaks after Abdominal Endovascular Aneurysm Repair. J. Clin. Med..

[4] Mannacio V.A., Mannacio L., Antignano A., Monaco M., Mileo E., Pinna G.B., Giordano R., Mottola M., Iannelli G. (2021). Status of coronary disease and results from early endovascular aneurysm repair after preventive percutaneous coronary revascularization. J. Card. Surg..

[5] Malas M., Arhuidese I., Qazi U., Black J., Perler B., Freischlag J.A. (2014). Perioperative mortality following repair of abdominal aortic aneurysms: Application of a randomized clinical trial to real-world practice using a validated nationwide data set. JAMA Surg..

[6] Hołda M.K., Iwaszczuk P., Wszołek K., Chmiel J., Brzychczy A., Trystuła M., Misztal M. (2020). Coexistence and management of abdominal aortic aneurysm and coronary artery disease. Cardiol. J..

[7] Baguet J.P., Chavanon O., Sessa C., Thony F., Lantelme P., Barone-Rochette G., Mallion J.M. (2012). European Society of Hypertension scientific newsletter: Hypertension and aortic diseases. J. Hypertens..

[8] Assania R., Daulay E., Hasan R. (2023). Association of Risk Factors for Coronary Artery Disease with The Incidence of Abdominal Aortic Calcification On Abdominal CT-Scan Imaging in H. Adam Malik General Hospital. J. Soc. Med..

[9] Nardi P., Rinaldi V., Costanzo M.L., Pasqua R., Loiacono F., Palumbo P., Miraldi F., Tanzilli G., D’Andrea V., Illuminati G. (2024). Simultaneous Percutaneous Coronary Intervention (PCI) and Endovascular Aneurysm Repair (EVAR): A Preliminary Report. J. Clin. Med..

[10] Tang Y., Shu C., Luo M., Fang K., Ye S., Yang M., Fan B., Xue Y., Zhao J., Chen Z. (2021). Mid-term results of one-stop endovascular aortic repair/percutaneous coronary intervention hybrid procedure for patients with aortic and coronary artery diseases. Vasc. Investig. Ther..

[11] Weng C., Wang J., Zhao J., Ma Y., Huang B., Yang Y., Yuan D., Wang T., Chen X. (2023). Fast-Track Management of Concurrent Percutaneous Coronary Intervention in Patients Scheduled for Endovascular Abdominal Aortic Aneurysm Repair. J. Endovasc. Ther..

[12] Williams A.M., Watson J., Mansour M.A., Sugiyama G.T. (2016). Combined Coronary Artery Bypass Grafting and Abdominal Aortic Aneurysm Repair: Presentation of 3 Cases and a Review of the Literature. Ann. Vasc. Surg..

[13] Okada K., Omura A., Kano H., Ohara T., Shirasaka T., Yamanaka K., Miyahara S., Sakamoto T., Tanaka A., Inoue T. (2012). Short and midterm outcomes of elective total aortic arch replacement combined with coronary artery bypass grafting. Ann. Thorac. Surg..

[14] Inciura D., Benetis R. (2006). Simultaneous coronary artery bypass grafting and vascular operations: Early and mid-term results. J. Cardiovasc. Surg..

[15] Endo M., Aomi S., Tomisawa Y., Uchikawa S., Kihara S., Yamasaki K., Nishida H., Kurosawa H. (2003). Selection of surgical strategy for abdominal aortic aneurysm coexisting with coronary artery disease; one-stage versus two-stage, and off-pump versus on-pump. Kyobu Geka.

[16] Pecoraro F., Wilhelm M., Kaufmann A.R., Bettex D., Maier W., Mayer D., Veith F.J., Lachat M. (2015). Early endovascular aneurysm repair after percutaneous coronary interventions. J. Vasc. Surg..

[17] Luo M.Y., Tang Y., Fang K., Chen Z., Chen L., Lu B., Chang Q., Sun X., Ouyang C., Shu C. (2016). “One-stop” endovascular treatment for concomitant coronary heart disease and aortic atherosclerotic disease. China J. Gen. Surg..

[18] van Herpt T.T.W., Timmermans S.A.M.E.G., van Mook W.N.K.A., van Bussel B.C.T., van der Horst I.C.C., Maessen J.G., Natour E., van Paassen P., Heuts S. (2022). Postsurgical Thrombotic Microangiopathy and Deregulated Complement. J. Clin. Med..

[19] Mejia E.J., Lin K.Y., Okunowo O., Iacobellis K.A., Matesanz S.E., Brandsema J.F., Wittlieb-Weber C.A., Katcoff H., Griffis H., Edelson J.B. (2022). Health Care Use of Cardiac Specialty Care in Children with Muscular Dystrophy in the United States. J. Am. Heart Assoc..

[20] Landesberg G., Mosseri M., Wolf Y.G., Bocher M., Basevitch A., Rudis E., Izhar U., Anner H., Weissman C., Berlatzky Y. (2003). Preoperative thallium scanning, selective coronary revascularization, and long-term survival after major vascular surgery. Circulation.

